# Molecular Characterization and Subtyping of *Blastocystis* Species in Irritable Bowel Syndrome Patients from North India

**DOI:** 10.1371/journal.pone.0147055

**Published:** 2016-01-19

**Authors:** Rojaleen Das, Shehla Khalil, B. R. Mirdha, Govind K. Makharia, Siddharta Dattagupta, Rama Chaudhry

**Affiliations:** 1 Department of Microbiology,All India Institute of Medical Sciences, New Delhi, India; 2 Department of Gastroenterology and Human Nutrition, All India Institute of Medical Sciences, New Delhi, India; 3 Department of Pathology, All India Institute of Medical Sciences, New Delhi, India; University Hospital Llandough, UNITED KINGDOM

## Abstract

*Blastocystis* species has been extensively studied in recent few years to establish its pathogenecity. Present study was designed to identify and examine the association of *Blastocystis* sp. and its subtypes with Irritable Bowel Syndrome (IBS).*Blastocystis* sp. detected using wet-mount microscopy, trichrome staining, *in-vitro* culture and Polymerase Chain Reaction (PCR) assay in a cohort of IBS patients (n = 150) and healthy controls (n = 100). Isolates of *Blastocystis* sp.were subtyped using Sequence Tagged Site and representative samples were sequenced at SSUrRNA locus.A total of sixty five isolates of *Blastocystis* sp. were identified [IBS (n = 50); Controls (n = 15)] of which 91% belonged to ST3 and 9% belonged to ST1. No other subtypes could be identified. Statistically significant association was observed between *Blastocystis* sp. and IBS patients; however no particular subtype could be ascertained to any particular clinical type of IBS.The frequency of occurrence of *Blastocystis* sp. was more in IBS patients as compared to the controls and ST3 being the most prevalent subtype. The genetic polymorphism of SSU-rRNA gene amongst the different *Blastocystis* sp.isolates found in this study reinforces the fact that these organisms are genetically highly divergent.

## Introduction

*Blastocystis* species,the non-motile Stramenopile, isolated from human gut [1] has four different morphological forms i.e. vacuolar, granular, ameboid and cystic. Among these, vacuolar is the most commonly isolated from human stool specimens. In less than a decade, 17 different subtypes (STs) of *Blastocystis* sp. have been described on the basis of Sequence Tagged Sites (STS) analysis on SSU-rRNA locus [2]. Subtypes ST1 to ST9 have so far been isolated from humans [1], and ST3 being the commonest subtype. Role of *Blastocystis* sp. as an etiology of gastrointestinal disorder has been linked to symptoms like chronic diarrhea, skin lesion and functional gastrointestinal disorder i.e. Irritable Bowel syndrome (IBS)which manifests with symptoms of recurrent abdominal pain associated with changes in bowel habit [3,4] Prevalence of *Blastocystis* as a causal agent of IBS varies with geographical region ranging from 2.6% to 100% [2,5,6].

In Indian subcontinent, clinical studies on IBS so far have been reported in only two studies with a prevalence of 4% to 4.2% [7,8]. Although four symptom-based clinical subgroups of IBS, such as IBS-C for patients with constipation, IBS-D for patients with diarrhea, IBS-M for patients with alternating diarrhea and constipation and IBS-U for patients with unsubtyped IBS have have been characterized, causal association of different subtypes of *Blastocystis* sp. in IBS patients was difficult to correlate in this part of the world.

The present prospective study was conducted with an aim to find out the causal association of *Blastocystis* species with IBS and further subtyping these isolates to extrapolate subtype association with that of different clinical types of IBS.

## Materials and Methods

### Study design

The present study was a hospital based observational study conducted from December, 2012 to October 2014 at All India Institute of Medical Sciences (AIIMS), a tertiary care health centre.

### Patients

Study population (cases) comprised of adult patients satisfying Rome III diagnostic criteria [9] for clinical diagnosis of IBS. A thorough clinical examination and investigation was carried out in all suspected IBS patients and sigmoidoscopy was performed in some to rule out Tuberculer colitis and Inflammatory Bowel disease (IBD).The enrolled patients were further examined and assessed for various factors such as stress disorders, particular food habits, menstrual history and any family history as these are known precipitating factors for IBS. Patients’ positive for infectious agents like *Salmonella*, *Shigella*, *Campylobacter jejuni* and gastrointestinal viruses were excluded from the study.Stool samples obtained from all the enrolled individuals were subjected to wet mount microscopy and samples positive for parasites like *Giardia intestinalis*, *Entamoeba dispar/histolytica*, *Dientamoeba fragilis and Endolimax nana* were excluded from the study. It was also ensured that patients who had a history of taking any anti parasitic drugs three weeks prior to sample collection were not included in the study.

A total of one hundred and fifty (n = 150) adult patients with clinical definition of IBS were enrolled. On the basis of clinical symptoms these patients were further categorized into IBS-D, IBS-C, IBS-M and IBS-U.Individuals above 18yrs of age who had no overt clinical signs and symptoms and any other gastrointestinal disorders after thorough clinical investigations were included as controls (n = 100).

### Clinical Samples

Three stool samples on three consecutive days were obtained from both cases and controls. Stool samples were first examined by direct microscopy using both saline and iodine wet-mount preparation.Faecal smears were also prepared and were stained with both Trichrome stain [10] as well as modified acid-fast stain [11] *In vitro* culture was simultaneously done for all stool samples using Modified Jone’s media [12] and were subcultured at interval of 3 and 5 days. The culture media was kept at least for 10 days before reporting the culture as negative for *Blastocystis sp*.

### DNA Extraction and PCR assay

Genomic DNA was extracted from the stool samples of [cases (n = 150) and controls (n = 100)] using Qiagen DNA stool minikit as per manufacturer’s instructions. For molecular detection of *Blastocystis sp*. Single round Polymerase Chain Reaction (PCR) assay was carried out using SSU-rDNA as the target gene.BhRDr (5’-GAG CTT TTT AAC TGC AAC AAC G-3’) and RD5 (5’-ATC TGG TTG ATC CTG CCA GTA-3’) were used as forward and reverse primer[13]. Subtyping of *Blastocystis sp*. was carried out based on Sequence Tagged site (STS) of *SSU rRNA* gene as described by Yoshikawa et al[14].

### Sequencing

Representative samples of *Blastocystis sp*.obtained from both cases as well as from controls were sequenced at SSU rRNA locus using Big Dye Terminator Chemistry in an automated sequencer ABI prism 310. DNA chromatograms were examined using the BioEdit software versions 7.1.3. The forward and reverse sequences were aligned pair-wise using Clustal W software and were manually refined to obtain a better consensus sequence. The sequences were analysed for subtypes as well as alleles using http://www.pubmlst.org/blastocystis database. Reference sequences from Genbank were used for phylogenetic analyses. The phylogenetic tree was constructed using MEGA6 software. Twenty (n = 20) representative isolates of *Blastocystis* sp. both from cases (n = 14) and controls (n = 6) were deposited in Genbank (Accession no.KP698119 to KP698138) (Table 1).

**Table 1:** *Blastocystis sp*. isolates from IBS patients from the present study and their Genbank Accession numbers.

Isolate	Host	Accession no.	Subtype	Allele
BH11	Human	KP698119	ST3	34
BH28	Human	KP698120	ST3	34
BH29	Human	KP698121	ST3	36
BH49	Human	KP698122	ST3	36
BH13	Human	KP698123	ST3	36
BH68	Human	KP698124	ST3	34
BH71	Human	KP698125	ST3	34
BH65	Human	KP698126	ST3	36
BH72	Human	KP698127	ST3	34
BH75	Human	KP698128	ST3	36
BH88	Human	KP698129	ST3	36
BH47	Human	KP698130	ST3	34
BH55	Human	KP698131	ST3	36
BH66	Human	KP698132	ST3	36
BH36	Human	KP698133	ST1	4
BH23	Human	KP698134	ST1	4
BH51	Human	KP698135	ST1	4
BH58	Human	KP698136	ST1	4
BH59	Human	KP698137	ST1	4
BH61	Human	KP698138	ST1	4

### Ethical

The study was ethically approved by institutional Ethical committee of All India Institute of Medical Sciences. Both informed and written consents were obtained from the patients and controls.

### Statistical analysis

Statistical analysis was done using STATA 12.2 software and wherever applicable p value, Odds Ratio and 95% confidence interval was calculated.

## Results

### Cases and Control cohorts

A total of one hundred fifty (n = 150) cases with a clinical diagnosis of IBS including ninety eight (n = 98) males and fifty two (n = 52) females were enrolled. Controls included eighty six (n = 86) males and fourteen (n = 14) females. The mean age of IBS patients and controls was 38 ± 10.5 years and was 33± 8.7 years, respectively. IBS patients were further categorized to different clinical subtypes. A total of 43% (64/150) belonged to IBS-M type whereas 42% (63/150),14% (21/150), and only 1% (2/150) belonged to IBS-D, IBS-C and IBS-U clinical subtypes respectively.

### Study of *Blastocystis* sp.in clinical samples

**S**tool wet-mount microscopy showed the presence of *Blastocystis* sp. in 32% (48/150) of IBS patients compared to 13% (13/100) in the controls. In contrast, modified Trichrome stained smears were positive for *Blastocystis* spp in 23.7% (42/150) of IBS patients and 8% (8/100) in controls. *In-vitro* culture showed a positivity of *Blastocystis* in 30% (45/150) and 2% (2/100) of the IBS patients and controls respectively. However, PCR assay showed positive amplification for *Blastocystis sp*. 33.3% (50/150) in case of IBS patients and 15% (15/100) in the control group (Table 2; S1 Table).

**Table 2:** Comparison of laboratory techniques for detection of *Blastocystis* sp.

	Wet mount Microscopy	Trichrome staining	*In vitro* culture	PCR assay
IBS patients(n = 150)	48 (32%)	42(28%)	45(30%)	50(33.34%)
Controls (n = 100)	13(13%)	8(8%)	3(3%)	15(15%)

**Fig 1:**
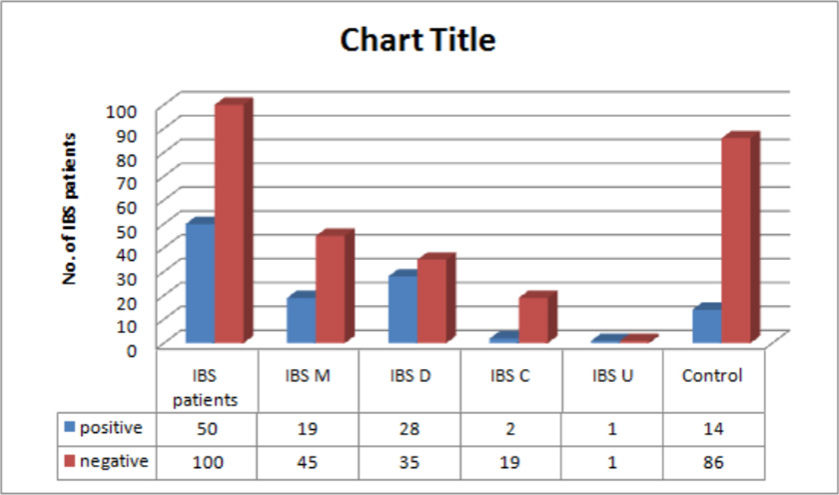
Distribution of *Blastocystis* sp in clinical types of Irritable Bowel Syndrome.

Fifty (n = 50) IBS patients positive for *Blastocystis sp*.were further analyzed for various clinical subtypes. IBS-D (55%, 28/50)was the most common clinical subtype in patients positive for *Blastocystis* spp.followed by IBS M (39.2%), IBS C (4%) and IBS U type (2%)(Fig 1; S1 Table).

### Subtyping of *Blastocystis* sp.

All sixty five (n = 65; cases = 50, control = 15) samples positive for *Blastocystis sp*.by PCR assay were further subtyped. ST 3 was the most common subtype identified in both IBS patients (47/50) as well as in controls (12/15) followed by ST1 (three in both cases and controls) by PCR assay (Table 3; S1 Table). Hence, a total of fifty nine (n = 59) isolates (47 from IBS patients and 12 from controls) belonged to ST3 and only six isolates (3 from IBS patients and 3 from controls) belonged to ST1.

**Table 3:** Distribution of subtypes in IBS patients and Control group.

Subtype	IBS patients (n = 50)	Controls (n = 15)
ST1	3 (6%)	3 (20%)
ST3	47 (94%)	12 (80%)

The frequency of occurrence of ST3 in IBS M and IBS D was 94% (18/19) and 92% (26/28), respectively, followed by ST1 (Table 4; S1 Table). *Blastocystis* from both IBS C and IBS U belonged to ST3.

**Table 4:** Distribution of *Blastocystis sp* subtypes in clinical types of IBS.

IBS Type	ST1	ST3
IBS M (n = 19)	1 (6%)	18 (94%)
IBS D (n = 28)	2 (8%)	26 (92%)
IBS C (n = 2)	-	2 (100%)
IBS U (n = 1)	-	1 (100%)
Total	3	47

### Sequence analyses

**Fig 2:**
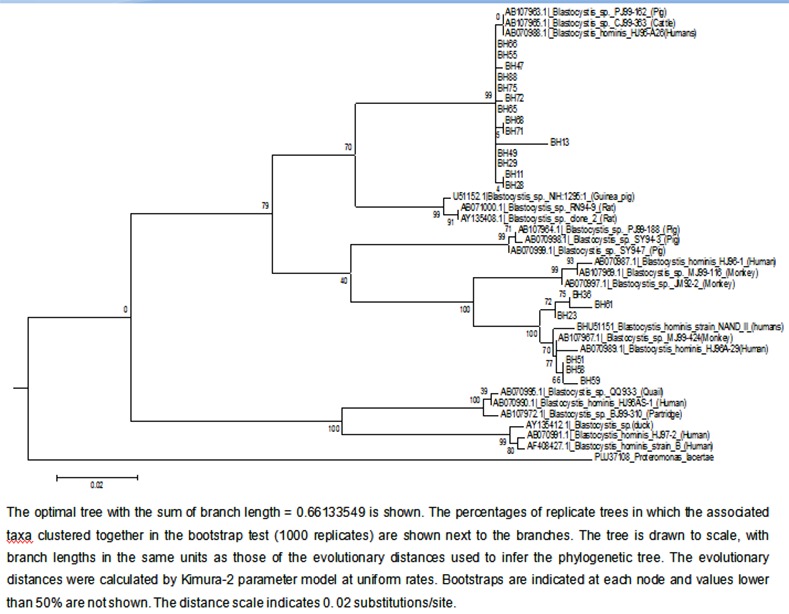
Phylogenetic tree of *Blastocystis sp* based on SSUrRNA gene sequence.

A phylogenetic analysis was done using 21 reference sequences [15] (Table 5). Phylogenetic tree was constructed using neighbor-joining (NJ) and was rooted using *Proteromonas lacertae* as an outgroup (Fig 2). *P*. *lacertae* was chosen as an outgroup since it has shown relationship with *Blastocystis sp*. in earlier phylogenetic studies [16]. Clusters in the phylogenetic trees were also considered if these had bootstrap support of greater than 50%. Only 20 *Blastocystis sp*. isolates were randomly chosen and were sequenced at SSU-rRNA locus. Amongst these 14 were subtyped as ST3 and 6 were subtyped as ST1. It was also seen that all ST1 isolates belonged to allele 4, however intra strain variation was observed in ST3. Amongst the 14 ST3 isolates, 8 belonged to allele 36 and 6 belonged to allele 34.

**Table 5:** GenBank reference sequences used in the construction of phylogenetic tree.

S. No.	Host	Accession No.
1.	Human	AB070989
2.	Human	AB070987
3.	Human	AB070997
4.	Human	AB070988
5.	Human	AB070990
6.	Human	AB070991
7.	Human	AF408427
8.	Monkey	AB107967
9.	Monkey	AB070997
10.	Monkey	AB107969
11.	Pig	AB107963
12.	Pig	AB070999
13.	Pig	AB107964
14.	Pig	AB070998
15.	Rat	AB071000
16.	Rat	AY135408
17.	Partridge	AB107972
18.	Quail	AB070995
19.	Duck	Y135412
20.	Cattle	AB107965
21.	Guinea pig	U51152

## Discussion

*Blastocystis* sp.have been established as a probable causal agent in patients suffering from IBS [5], however, the exact pathogenic mechanisms have not been clearly delineated, except some protease enzymes of *Blastocystis* sp. that cause mucosal disruption and dysbiosis [17]. Most of the studies related to association of *Blastocystis* sp.in IBS cohorts have been conducted in Middle-East, Southeast Asia and South-America.

In the present study amongst IBS patients, *Blastocystis sp*.could be detected at a frequency of 32% (48/150), 33.34% (50/150), 28% (42/150) and 30% (45/150) using wet-mount, PCR assay, modified trichrome stain and *in-vitro* culture respectively. In this study, sensitivity of microscopy was more than *in-vitro* culture, which is in contrast to studies reported by Tan and Dogruman[1,18]. Relatively higher sensitivity of microscopy in the present study can be attributed to examination of three consecutive stool samples on three consecutive days. Concerning the importance of detection of the *Blastocystis* sp.and PCR being an objective method has been advocated as the method of choice for the detection [19].

*Blastocystis sp*.could be detected at a frequency of 33.34% (50/150) and 15% in cases and controls respectively and this difference was statistically significant [p = 0.0016; OR = 2.833; 95% CI = 1.48 to 5.40]. Studies from Turkey [12] Pakistan [20] and Italy [21]have also reported significant association of *Blastocystis sp*. in IBS patients compared to healthy controls. Nevertheless, contrasting results from Thailand [22] and Mexican studies [23] have shown no difference in the frequency of *Blastocystis* sp isolation between IBS patients and the controls

*Blastocystis* sp.from IBS patients in the present study suggests the possible causal role for IBS. Among fifty IBS patients positive for *Blastocystis* sp, 56% (28/50) belonged to IBS-D followed by 38% (19/50) IBS-M and 4% (2/50) IBS-C type.Upon statistical analysis a significant association between *Blastocystis* sp. and IBS-D could be made (p = 0.015; OR = 2.36 with 95% CI of 1.16 to 4.72) but not with other clinical subtypes of IBS.Using conventional PCR assay, Yakoob et al. [20], had reported a higher prevalence of *Blastocystis sp*. in an IBS-D clinical subtypes in Pakistan (44% versus 21% in controls, p<0.001). On the contrary, study by Nourrisson et al.,[24] from France, did not show any The disparity in prevalence of *Blastocystis sp*.in each clinical subtype of IBS may possibly be explained by the number of IBS patients enrolled in the respective studies.

*Blastocystis* sp.subtype ST 3 was the most common subtype (92%) in the present study. According to the recent study from India [25], ST3 subtype was found in all 27 normal healthy individuals positive for *Blastocystis* sp.and only in 2 samples showed mixed infection of both ST1 and ST3 In one of the comprehensive study by Yoshikawa et al., [26] ST3 was the dominant subtype in the four different populations from different countries that included Japan, Bangladesh, Pakistan, and Germany, with a frequency ranging from 41.7% to 92.3%. Study from Columbia has also shown marked association of ST1 being commonly isolated from asymptomatic individuals, ST2 from patients presenting with diarrhea and ST3 exclusively in patients with IBS [27] The difference in the relative diversity and prevalence of subtypes of *Blastocystis* sp. probably reflects epidemiological and demographic differences including climatic conditions, geographical attributes, cultural habits, proximity and exposure to reservoir hosts, and mode of transmission [28] Further, ST3 was relatively common with IBS D (56%, 26/47) followed by IBS M (38%, 18/47) and IBS C (4%, 2/47). Subtype association of *Blastocystis* sp in IBS patients was not statistically significant [p = 0.7; OR = 0.61; 95% CI = 0.05 to 7.30]. In contrast, a study from neighbouring country Pakistan showed association between ST1 and IBS-D clinical subtype [20].

Phylogenetic studies of *Blastocystis* sp. based on SSU rRNA [14,16,29,30]) suggested low host specificity of *Blastocystis sp*. and also the zoonotic potential of the parasite. Molecular phylogenetic study was done to determine the extent to which the parasite can be transmitted among host species and the potential reservoir for infection in humans.

All the *Blastocystis* sp. SSUrDNA sequences included in the analysis were edited to include only the region under study, and phylogenetic analysis was based on 540 bp barcode region located on SSUrRNA locus, since the complete SSU-rDNA sequence of *Blastocystis* sp. is not required for assignment of an isolate to its appropriate subtype [13]. Alignment of SSUrRNA sequences of our isolates were compared with those of already reported *Blastocystis* sp. isolates from human and animal origin and it showed two different clades with a good bootstrap support. Minor differences in the branch order among the subgroups were observed. Isolates subtyped as ST3 (n = 14) were clustered together along with *Blastocystis* sp. PJ99-162 isolate from pig, *Blastocystis* sp. CJ99-363 isolate from cattle and *Blastocystis* sp. isolate from humans. Similary isolates subtyped as ST1 formed a cluster with *Blastocystis* sp. Strain NAND II from humans, *Blastocystis* sp. MJ99-424 from monkeys and *Blastocystis* sp. HJ96A-29 from humans. The phylogenetic tree shows that the same subtypes identified in the present study clustered together, where as the two subtypes clustered as two independent monophyletic groups. Isolate BH13 showed sequence variation at positions 47 to 49 and 52 to 56, and it formed a separate branch in the ST3 cluster of the phylogenetic tree.

Hereby it was clear that *Blastocystis* sp. subtypes 1 and 3 were shared by isolates from varied hosts and its cross-infectivity among different hosts. The findings clearly show that close association between animals and humans isolates exists, and that the animals can facilitate the transmission of *Blastocystis* sp. This has been showed by previous studies [16] also that no exclusive ‘human’ clade seems to exist because sequences from human isolates are present in all clades

## Conclusion

To best of our knowledge, this is the first molecular study showing correlation of *Blastocystis* sp. and IBS patients from India. The frequency of occurrence of *Blastocystis* sp. was highest in IBS-D clinical subtype. The genetic polymorphism of SSU-rRNA gene amongst the different *Blastocystis sp*.isolates found in this study revealed that these organisms are genetically highly divergent. Among the two identified subtypes, ST3 was predominant. The pathogenic potential of ST3 could not be elucidated in the present study and further studies in this regard will extrapolate various unresolved issues.

## Supporting Information

S1 Table IBS Patients clinical details and laboratory findings. (XLSX)
